# Multicellular Crosstalk Between Exosomes and the Neurovascular Unit After Cerebral Ischemia. Therapeutic Implications

**DOI:** 10.3389/fnins.2018.00811

**Published:** 2018-11-06

**Authors:** Ana-Maria Zagrean, Dirk M. Hermann, Ioan Opris, Leon Zagrean, Aurel Popa-Wagner

**Affiliations:** ^1^Division of Physiology and Neuroscience, Carol Davila University of Medicine and Pharmacy, Bucharest, Romania; ^2^Department of Neurology, Chair of Vascular Neurology, Dementia and Ageing Research, University Hospital Essen, Essen, Germany; ^3^Center of Clinical and Experimental Medicine, University of Medicine and Pharmacy of Craiova, Craiova, Romania; ^4^Department of Neurological Surgery, University of Miami, Miami, FL, United States; ^5^School of Medicine, Griffith University, Gold Coast, QLD, Australia

**Keywords:** exosome, nanovesicles, neurovascular unit, blood-brain barrier, miRNA, stroke, mesenchymal stem cells

## Abstract

Restorative strategies after stroke are focused on the remodeling of cerebral endothelial cells and brain parenchymal cells. The latter, i.e., neurons, neural precursor cells and glial cells, synergistically interact with endothelial cells in the ischemic brain, providing a neurovascular unit (NVU) remodeling that can be used as target for stroke therapies. Intercellular communication and signaling within the NVU, the multicellular brain-vessel-blood interface, including its highly selective blood-brain barrier, are fundamental to the central nervous system homeostasis and function. Emerging research designates cell-derived extracellular vesicles and especially the nano-sized exosomes, as a complex mean of cell-to-cell communication, with potential use for clinical applications. Through their richness in active molecules and biological information (e.g., proteins, lipids, genetic material), exosomes contribute to intercellular signaling, a condition particularly required in the central nervous system. Cerebral endothelial cells, perivascular astrocytes, pericytes, microglia and neurons, all part of the NVU, have been shown to release and uptake exosomes. Also, exosomes cross the blood-brain and blood-cerebrospinal fluid barriers, allowing communication between periphery and brain, in normal and disease conditions. As such exosomes might be a powerful diagnostic tool and a promising therapeutic shuttle of natural nanoparticles, but also a means of disease spreading (e.g., immune system modulation, pro-inflammatory action, propagation of neurodegenerative factors). This review highlights the importance of exosomes in mediating the intercellular crosstalk within the NVU and reveals the restorative therapeutic potential of exosomes harvested from multipotent mesenchymal stem cells in ischemic stroke, a frequent neurologic condition lacking an efficient therapy.

## Introduction

At the interface with the bloodstream, neurovascular units (NVUs) are structural and functional multicellular modules consisting of neurons, perivascular astrocytes, microglia, pericytes, extracellular matrix and the endothelial cells of the brain microcirculation. They provide a coordinated neurovascular coupling and maintain a highly selective blood-brain barrier (BBB) (Abbott, 2002). The dynamic multicellular crosstalk within the NVUs in physiological and pathological conditions could reveal novel cell-targeted therapeutic strategies with impact on the BBB, cerebral homeostasis and brain functions (Attwell et al., 2010; Abbott and Friedman, 2012).

The endothelial cells of the BBB are interconnected by tight and adherens junctions and form a continuous layer. This layer selectively buffers the impact of fluctuations in blood composition on brain interstitial fluid, regulating the brain microenvironment and neuronal signaling (Abbott, 2013). Various transcellular transport systems across the BBB have been described, as carrier mediated transport, receptor-mediated transport, ion transfer, efflux carriage, adsorptive-mediated passage, and fluid-phase endocytosis (Zlokovic, 2008).

Apart from the classical modes of intercellular communication, such as ligand-receptor interactions, direct cell-cell contacts (e.g., gap junctions) or paracrine signaling (Goodenough et al., 1996), a significant experimental evidence has confirmed that several physiological and pathophysiological processes are controlled by the extracellular membrane vesicles, such as exosomes and microvesicles, secreted from various cellular sources into the body fluids and interconnecting cells without direct cell-to-cell contact (Valadi et al., 2007). This type of signaling occurs mainly through exosomes, which are nano-sized vesicles that easily transfer biological information from cell to cell. This is achieved by means of exosomal molecules that would usually not cross membrane barriers. This shows the capability of inducing functional changes in target cells and modulating local and systemic crosstalk (Krämer-Albers and Hill, 2016). In the brain, exosomes are released from all types of cells (Frühbeis et al., 2013) and are bidirectionally transported through the blood-brain communication interfaces, blood-brain and blood-cerebrospinal fluid barriers (Balusu et al., 2016). These blood-brain interfaces are potential pathways for therapeutically administered exosomes.

Given their capacity to easily reach body compartments and connect origin cells with target cells, exosomes have a promising potential to be used in clinical applications. Indeed, exosomes have shown the capacity to serve both as biomarkers and novel therapeutic tools in the nervous system pathologies lacking efficient therapies, such as stroke (Barile and Vassalli, 2017). The cellular interactions within NVU might contribute to (i) the restoration of a well-organized cerebral microvasculature by providing trophic support and a stimulating brain microenvironment (Hermann and ElAli, 2012), and (ii) the remodeling of parenchymal tissue, including axonal sprouting, dendritic growth and synaptic reorganization (Hermann and Chopp, 2012). However, there is still more to explore about the diagnostic benefits and therapeutic roles of exosomes, their production, release, transport, uptake, signaling potential, change of their cargo proteins profile and miRNAs (Zhang and Chopp, 2016). Here, we review the roles of exosomes in mediating the intercellular crosstalk within the NVU and the therapeutic potential of exosomes derived from multipotent mesenchymal stem cells (MSCs) in stroke.

## Exosomes’ as a Biological Communication Tool

Exosomes are defined as 30–100 nm sized membrane vesicles derivatives of the endosomal compartment and correspond to the intraluminal vesicles of multivesicular bodies (MVBs) that upon fusion of the MVBs with the plasma membrane are released as exosomes into the extracellular environment (Lener et al., 2015), where they act as signaling organelles for intercellular communication. From the extracellular milieu, exosomes may contact target cells by (i) receptor-mediated adhesion to the cellular plasma membrane, followed by endocytic uptake and internalization, (ii) direct fusion of the exosome membrane with the target cell membrane and subsequent exosomal content release into the recipient cell (Bang and Thum, 2012).

Exosomes’ vesicles are homogenous in shape, surrounded by a phospholipid membrane displaying membrane proteins, such as cell-specific receptors, and containing cell-type specific combinations of lipids, metabolites, coding and non-coding RNAs (miRNA, sRNA), single- and double stranded DNA, cytosolic and membrane proteins including enzymes, growth factors, receptors and cytokines (Théry et al., 2001; Lener et al., 2015). Exosomal lipids (e.g., phosphoglycerides, sphingomyelin, cholesterol, ceramide) are important for providing structural stability. Proteins of the exosomes are characteristic for their endosomal origin, and include membrane transport and fusion proteins (annexins, flotillin), proteins involved in cell targeting (tetraspanins, mostly CD9 and CD63) or other proteins correlated with their biogenesis from MVBs, as the tumor susceptibility gene 101 (TSG101) (András and Toborek, 2016). Exosomes also contain heat-shock proteins (Hsp60, Hsp70, Hsp90), known for their neuroprotective potential. Also, they expose low levels of phosphatidylserine and cell-type-specific proteins. One of the most important function of the exosomes is targeting cellular pathways in the recipient cells through their RNAs and miRNAs cargo (Ling et al., 2013).

**FIGURE 1:**
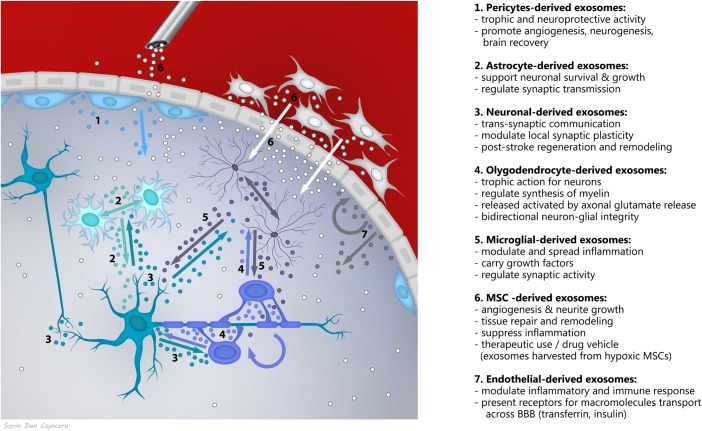
Exosomes as a fundamental mechanism of communication between components of the neurovascular unit.

Novel research supports exosomes as a fundamental mechanism of communication in the nervous system, with roles in brain homeostasis and plasticity (Holm et al., 2018), acting as bidirectional cargo in brain-periphery communication and within the brain, in between neurons, glia, vascular and perivascular cells (Figure 1). Exosome secretion has been described from (i) depolarized/stimulated cortical neurons, mainly from the somato-dendritic compartments (Faure et al., 2006; Lachenal et al., 2011; Von Bartheld and Altick, 2011), (ii) oligodendrocytes (Frühbeis et al., 2013), (iii) microglia (Potolicchio et al., 2005), (iv) astrocytes when activated by oxidative and heat stress (Taylor et al., 2007), (v) endothelial cells (Dozio and Sanchez, 2017), and (vi) pericytes (Mayo and Bearden, 2015), known to generate MSCs in the perivascular area of the lesioned or inflamed vessels (Caplan, 2008; Caplan, 2016).

The complex and versatile exosomal signaling was shown to impact the synaptic activity (e.g., neuronal origin exosomes exhibiting neurotransmitter receptors bind neurotransmitters within the synapse to stop signaling), *trans*-synaptic communication, synaptic plasticity, maintenance of myelination, angiogenesis, neurovascular integrity, but also on neuroregeneration and neuroprotection in response to disease conditions (Holm et al., 2018). For example, angiogenesis could be stimulated both by activation of signaling pathways PI3K, ERK1/2, Wnt4/ß-catenin or NF-kB and transfer of the transcription factors STAT3, STAT5, transfer of lipids like S1P, transfer of proteins including VEGF, FGF-2, PDGF, metalloproteases, but also by the transfer of micro-RNA-126, miR-214, miR-296, and miR-150 (for a review, see Todorova et al., 2017). Likewise, after experimental stroke, treatment with exosomes isolated from miR-133b-overexpressing MSCs, significantly increased functional improvement and neurite remodeling/brain plasticity in the ischemic boundary area compared with control animals (Xin et al., 2017b). Recently, it was also reported that miR-26a is a physiological regulator of mammalian axon regeneration by targeting glycogen synthase kinase 3β (GSK3β) in adult mouse sensory neurons *in vitro* and *in vivo* (Tsenkina et al., 2015).

Exosomes can also propagate inflammation across the BBB and within the brain, as brain endothelial cells activated by systemic inflammation further activate the neighboring cells in the NVU via secreted exosomes (Balusu et al., 2016; Holm et al., 2018). The pathogenic role of microglia-derived exosomes in the inflammatory response was demonstrated in a model of traumatic brain injury (TBI) i.e., *in vitro* activated microglia-derived exosomes induced neuroinflammation at the site of injection and around the lesion. Furthermore, circulating enriched exosomes or CD11b-isolated microglia from the TBI brain *ex vivo*, initiated neuroinflammation following intracortical injection in naïve animals (Verderio et al., 2012; Kumar et al., 2017). The pathogenic effects of microglia-derived exosomes could be mediated by pro-inflammatory mediators TNF-alpha, IL-1β and miR-155 (Kumar et al., 2017).

Also, exosomes contribute to disease spreading by acting like Trojan horses for neurodegenerative agents (e.g., toxins, such as tetanus toxin, protein aggregates, such as phosphorylated Tau, amyloid Aβ or synuclein) (Bellingham et al., 2012; Holm et al., 2018). Through their non-coding RNA cargo and miRNA transfer, exosomes are involved in epigenetic regulation of neuro-glial communication within the nervous system, but also in brain-body epigenetic interconnection (Lai and Breakefield, 2012).

## Neurovascular Unit Remodeling in Response to Stroke

Following the failure of acute neuroprotection therapies, major efforts are currently made worldwide to promote neurological recovery and brain plasticity in the subacute and post-acute phases of stroke. For over more than two decades, therapeutic efforts in the stroke field have focused on the promotion of neuronal survival, which failed to succeed in clinical trials in humans until now (Savitz and Fisher, 2007; Ginsberg, 2008). From failure to translate successful neuroprotection therapies from animal models to humans, it may be concluded that the stimulation of survival alone is without prospect, as long as no successful remodeling of brain tissue stimulated by a permissive microenvironment takes place. Indeed, studies done recently have shown that extensive remodeling occurs in the brain following an ischemic event (Hermann and Zechariah, 2009; Hermann and Chopp, 2012). Currently, there is hope that stroke recovery might be promoted through pharmacological or cell-based therapies. Indeed, promising results from experimental studies have led to clinical trials, the results of which are currently awaited (Lener et al., 2015).

Remodeling of ischemic brain tissue involves interactions between neurons, glial and microvascular cells that create a microenvironment in which neurological recovery may ensue. Neurons and brain capillaries sprout. Neuronal outgrowth enables the formation of functional axons and synapses in the brain both over long [e.g., along pyramidal tract (Andres et al., 2011; Reitmeir et al., 2011)] and short (e.g., within motor cortex (Clarkson et al., 2010; Hermann and ElAli, 2012) distances, thus allowing for the restitution of neuronal networks that were damaged by the stroke event. The remodeling of ischemic brain tissue also includes responses of immature cells, namely of endothelial progenitor cells (EPC), neural progenitor cells (NPC), and inflammatory cells. New blood vessels are formed, and EPC and NPC are attracted to the stroke lesion. Glial cells contribute to the remodeling of the extracellular matrix, enabling neuronal plasticity.

In the process of brain remodeling, proliferating microvascular cells play a supportive role, enabling the migration of neural precursor cells and promoting the remodeling of neurons and glial cells via secretion of growth factors (Hermann and Zechariah, 2009). This rearrangement of cell-cell interactions is followed by the recovery of the BBB, leading to the restoration of brain homeostasis (Hermann and ElAli, 2012).

The remodeling potential of the NVU serves as an important therapeutic target in stroke and other acute neurologic conditions. After stroke, damaged and inflamed endothelium release pro-inflammatory factors and extracellular vesicles (EVs) that pass through the leaky BBB and activate astrocytes and microglia to release pro-inflammatory cytokines (TNFα, IL1β) (Norden et al., 2014). Microglia also release the anti-inflammatory cytokine IL-10 that acts on reactive astrocytes to modify their cytokine secretion from a pro-inflammatory profile toward a pro-recovery one, represented mainly by TGFβ. During the post-stroke BBB repair and parenchymal remodeling process, NVU cells cooperate and release pro-recovery factors (e.g., IL-4, IL-10, TGFβ) that switch microglia into a pro-remodeling phenotype that release growth factors (Norden et al., 2014). Moreover, IL-10 acts on the endothelial and vascular cells to modulate vascular repair and remodeling, diminishes leukocyte–endothelial interactions, decreases expression and activation of cytokine receptors, promotes NO-induced vasodilatation and diminish ROS production and oxidative stress by inhibiting a NADPH oxidase subunit (Nox1) with impact on degenerative vascular remodeling (Dammanahalli et al., 2011; Garcia et al., 2017). Overall, IL-10 secreted from the NVU cells, but also from MSCs and their exosomes (Nakajima et al., 2017), is a pro-survival factor for neurons and glial cells that diminishes the post-lesional inflammatory response and limits the secondary damage during the resolution phase (Mosser and Zhang, 2008).

Pericytes behavior in different phases of ischemic stroke were recently described (Yang et al., 2017). Briefly, during the stroke hyperacute phase, pericytes constriction causes capillary occlusion (no-reflow phenomenon). Then, during the acute phase, pericytes have a pro-inflammatory and immune-modulatory action, with consecutive increase in BBB permeability and brain edema. By protecting the endothelium on its abluminal side and through release of neurotrophins, pericytes stabilize the BBB and protect brain parenchyma. Further, during post-stroke recovery phase, pericytes have a neuroprotective activity, promoting angiogenesis, neurogenesis, and brain recovery. The complex multifaceted, multistage pericytes intervention in ischemic-reperfusion injury and repair processes, recommend them for new targeted therapeutic strategies (Cai et al., 2017).

## Neurovascular Unit-Derived Exosomes in Response to Stroke

Pericytes are important players in post-stroke NVU remodeling. Thus, they were shown to become activated and gain multipotent stem cell phenotype after brain ischemia and express the neuroepithelial stem cell marker nestin, with a potential to differentiate into neural and vascular precursor lineages (Nakagomi et al., 2015). Cooperation between endothelial cells and pericytes occurs both through paracrine interaction, but also through an exosomal bidirectional communication and is essential for preserving the microvascular functionality and stability. For example, endothelium or pericyte-derived hypoxic exosomes were shown to induce an angiogenic program (Fan, 2014; Mayo and Bearden, 2015). Secondary to local injury and perivascular inflammation, MSCs are released from their perivascular location and secrete bioactive molecules and exosomes with immunomodulatory and trophic effects, supporting the regenerative microenvironment needed for the post-injury recovery (Caplan and Correa, 2011). A recent work even suggests that perivascular MSCs are adventitial cells, acting as precursors of pericytes and other stromal cells during tissue homeostasis (de Souza et al., 2016). Not all pericytes can generate MSCs. It has been recently shown that from the various sub-population of existing pericytes, not all of them can act like stem cells, and some act like fibroblasts.

Neuronal exosomes are present at synaptic level, both within pre- and postsynaptic compartments, and transport synaptic receptors (e.g., AMPA receptors, GPCRs) (Koniusz et al., 2016) contributing to synaptic plasticity, both locally and within broader neuronal networks (Chen and Chopp, 2018). The activity within glutamatergic synapses, which is increased in post-stroke excitotoxic conditions, stimulates neuronal release of exosomes that preferentially bind to adjacent neurons, impacting on interneuronal communication (Chivet et al., 2014). The exosomes released secondary to neuronal depolarization are rich in miRNAs, potentially promoting synaptic plasticity by enabling the rapid translation of associated proteins (Goldie et al., 2014).

The interactions between neurons, glial cells and microvascular cells are finely tuned. They involve mutual cell to cell communication via release of growth factors as well as physical cell-cell interactions across the extracellular matrix that is itself subjected to remodeling processes after stroke (Rosell and Lo, 2008). Considering the complexity of these systems and considering both the structural and functional heterogeneities of brain structures and the heterogeneities of ischemic strokes with regard to their size, etiology, and localization (Hermann and Chopp, 2012), the development of neurorestorative therapies is a true challenge (Hermann et al., 2015).

## The Potential Use of Mesenchymal Stem Cells and Their Exosomes for Stroke Therapy

Ischemic stroke is a leading cause of death and long-term disability in industrialized countries, with thrombolysis and interventional vascular recanalization being the only treatments available. Due to severe side effects and a narrow therapeutic time window, only a small proportion of stroke patients receive this therapy. Thus, additional therapeutic concepts are mandatory (Hermann and Chopp, 2012). Strategies that promote neuronal survival in the acute stroke phase have successfully been studied in experimental stroke models, but were not successful in clinical trials. Therefore, the research focus has recently shifted from the acute to post-acute stroke phase (Hermann and Chopp, 2012). After acknowledging that transplanted cells integrate poorly into existing neural networks and that they induce brain remodeling in a paracrine way by secreting a heterogeneous group of nanovesicles, these EVs have been identified as key players that mediate restorative effects of stem and progenitor cells in ischemic brain tissue. Neuroprotection as observed after EV infusion in experimental stroke models is related to stem cell application in stroke. As a matter of fact, stem cell-induced neurological recovery after stroke is not a consequence of cell regeneration but due to paracrine mechanisms of grafted cells, among which stem cell-derived EVs are key mediators (Doeppner et al., 2018).

Blood-brain barrier may block or diminish the access of therapeutic agents within the central nervous system and therefore many nervous system diseases lack an efficient treatment because of a deficient drug delivery vehicle. Considering this important issue, research is nowadays developing nanocarriers for brain targeted drug delivery and exploit the potential use of stem cells to secrete exosomes, as natural nanovesicles rich in biological active molecules. As lipid-bound nanoparticles, exosomes easily interconnect cells and cross selective-permeable membranes such as BBB, thus emerging as versatile tools for new therapeutic strategies (e.g., regenerative, immune-modulatory or anti-tumor therapies), either acting through their biochemically active constituents (e.g., proteins, lipids, genetic material), or serving as natural nonimmunogenic vehicles for drug delivery (Lener et al., 2015).

### Non-exosomal Effects of Mesenchymal Stem Cells

The human brain contains reservoirs of neural stem and precursor cells in the subventricular zone (SVZ) surrounding the lateral ventricles (Bacigaluppi et al., 2009). Although cerebral ischemia triggers the activation of these cells and promotes their migration toward ischemic lesion sites, their siblings hardly survive and differentiate within the ischemic milieu (Doeppner et al., 2012, 2014b). To improve brain remodeling and plasticity, and to bypass limitations of endogenous neurogenesis following ischemic stroke, a variety of approaches started to focus on the transplantation of NPCs or somatic stem cell entities, such as MSCs (Popa-Wagner et al., 2006, 2007, 2011). MSCs secretome comprises growth factors, cytokines, chemokines, extracellular matrix components, genetic material, but also EVs (exosomes and microvesicles), recommending them as versatile tools in clinical applications (Gaceb et al., 2018).

Mesenchymal stem cells, known as “sentinel and safe-guards of injury” (Caplan, 2016), were shown to produce neurotrophic factors such as nerve growth factor, brain-derived neurotrophic factor, or glial-derived neurotrophic factor, explaining their therapeutic potential (Lopatina et al., 2011).

Initially, it was assumed that transplanted NPCs and MSCs home to affected sites and, upon expansion and differentiation, directly replace the lost brain cells to restore tissue functions. In this context, our lab has comprehensively characterized the therapeutic effects of SVZ-derived adult NPCs in a mouse model of ischemic stroke, i.e., transient proximal (i.e., intraluminal) middle cerebral artery occlusion (MCAO). We observed that systemic NPC delivery induces profound brain tissue remodeling, reflected by reduced secondary neurodegeneration, reduced neuroinflammation, reduced astrogliosis and reduced microglial activation, that was associated with functional neurological recovery (Bacigaluppi et al., 2009; Doeppner et al., 2012, 2014a,b). Remarkably, it turned out that systemic intravenous administration of adult NPCs was more effective than intracerebral transplantation. Indeed, systemic administration effectively resulted in the stabilization of BBB integrity. However, just 0.1–0.3% of intravenously transplanted NPCs were detected in the brain and most of them were in an undifferentiated state (Bacigaluppi et al., 2009). These findings imply that NPCs act in a paracrine rather than a cellular mode.

### Exosomal Effects of Mesenchymal Stem Cells

The exosomes’ database ExoCarta^1^ reports more than 900 species of proteins associated with MSCs-derived exosomes, but recent data from proteomic analysis, identified more than 2000 proteins in MSC-exosome, many of them being involved in brain repair (Otero-Ortega et al., 2017). These were shown to increase glial production of anti-inflammatory and immuno-regulatory mediators, TGFβ1 and IL-10 (Burrello et al., 2016), with significant roles in NVUs’ recovery and remodeling. Also, it was recently shown that IL-10 is one of the neuroprotective factors through which transplanted MSCs act after an ischemic stroke. Thus, MSCs overexpressing IL-10 improved neuronal survival in the ischemic hemisphere (Nakajima et al., 2017). Interestingly, MSCs-derived exosomes were shown to exhibit post-stroke changes in their miRNA profile, mostly in the miRNAs actively involved in the repair process by altering gene expression and promoting brain recovery (Liu et al., 2013).

Classically, paracrine effects were thought to be mediated by soluble molecules such as growth factors, cytokines, chemokines and hormones. More recent data, however, demonstrate that several physiological and pathophysiological processes are controlled by exosomes (Cramer et al., 2017). In experimental stroke models, evidence was provided that exosomes exert neuroprotective, proangiogenic and neuronal plasticity-promoting functions (Xin et al., 2013). Thus, systemic administration of MSC-derived exosomes in a rat model of stroke improved functional recovery and enhanced neurite remodeling, neurogenesis, and angiogenesis (Xin et al., 2013). Furthermore, administration of combined xenogenic (from mini-pig) adipose-derived mesenchymal stem cell (ADMSC) and ADMSC-derived exosome therapy has been shown to reduce brain-infarct zone (BIZ) and enhance neurological recovery in rat after acute ischemic stroke (Chen et al., 2016). At molecular level the beneficial effects of MSC-derived exosomes could be mediated by the miR-17-92 cluster. Thus, rats subjected MCAO and treated with miR-17-92 cluster-enriched exosomes, performed significantly better than the control rats treated with MSC exosome alone (Xin et al., 2017a). Similarly, administration of exosomes isolated from miR-133b-overexpressing MSCs lead to increased neural plasticity and improvement of functional recovery after stroke in rats (Xin et al., 2017b).

Based on these observations, we performed a direct head-by-head comparison of the therapeutic effects of MSCs and their exosomes in a murine model of transient intraluminal MCAO, which predominantly affects the striatum and most lateral parts of the overlying cerebral cortex, showing that systemic MSCs and MSC-derived exosomes are equally effective in enhancing stroke-related motor and coordination recovery thereby confirming the beneficial effects of the exosome therapy reported by Chopp and colleagues who observed a significant reduction in neurological impairment that improved gradually over 4 weeks after systemic delivery of MSC-derived EVs (MSC-EVs) in a model of transient MCAO in rats (Xin et al., 2013). Both therapies promoted post-ischemic endogenous neurogenesis and angiogenesis and reversed the stroke-associated immunodepression (Doeppner et al., 2015).

## Conclusion and Perspectives

Ischemic stroke is a leading cause of death and long-term disability for which no restorative therapy is available. After stroke, the NVU is compromised and has become a major target for restorative therapies in the central nervous system. Emerging research has revealed that the nano-sized exosomes could be used for the NVU remodeling after stroke, due to their ability to mediate cell-to-cell communication. Considering the side effects typically attributed to cell-based therapies, in particular, malignant transformation of the transplanted cells, MSC-derived exosomes are attractive candidates for stroke therapy, as emphasized by a recent position paper (Lener et al., 2015). Indeed, systemic administration of MSC-derived exosomes is effective in enhancing stroke-related motor and coordination recovery in experimental stroke models, fueling the hope for clinical studies. Nevertheless, for clinical applications we need further studies to shed light on (i) mechanisms of the interaction between exosomes and target cells, (ii) circulation kinetics and biodistribution; (iii) biogenesis mechanism; (iv) potential side effects. For example, tumor-secreted exosomes may act as mediators in cancer metastasis by maintenance and enhancement of tumor microenvironment (Salido-Guadarrama et al., 2014; Cheng et al., 2017; Li et al., 2018). Likewise, several studies have reported high levels of cholesteryl ester (CE), triacylglycerol (TAG) and cardiolipin in exosomal preparations fueling concerns about increasing the risk of stroke instead of having a beneficial effect (Llorente et al., 2007; Van Meer et al., 2008; Strauss et al., 2010; Record et al., 2014; Skotland et al., 2017; Popa-Wagner et al., 2018).

Furthermore, considering that ischemic stroke mainly affects elderly patients, experimental data in aged rodents are urgently required before a clinical proof-of-concept study in human patients should be envisaged (Popa-Wagner et al., 2006, 2007, 2018; Balseanu et al., 2014).

## Author Contributions

All authors listed have made a substantial, direct and intellectual contribution to the work, and approved it for publication.

## Conflict of Interest Statement

The authors declare that the research was conducted in the absence of any commercial or financial relationships that could be construed as a potential conflict of interest.

## Footnotes

1 http://www.exocarta.org/

## References

[B1] Abbott N. J. (2002). Astrocyte–endothelial interactions and blood–brain barrier permeability. *J. Anat.* 200 629–638. 10.1046/j.1469-7580.2002.00064.x 12162730 PMC1570746

[B2] Abbott N. J. (2013). Blood–brain barrier structure and function and the challenges for CNS drug delivery. *J. Inherit. Metab. Dis.* 36 437–449. 10.1007/s10545-013-9608-0 23609350

[B3] Abbott N. J. Friedman A. (2012). Overview and introduction: the blood-brain barrier in health and disease. *Epilepsia* 53(Suppl. 6), 1–6. 10.1111/j.1528-1167.2012.03696.x 23134489 PMC3625728

[B4] András I. E. Toborek M. (2016). Extracellular vesicles of the blood-brain barrier. *Tissue Barriers* 4:e1131804. 10.1080/21688370.2015.1131804 27141419 PMC4836554

[B5] Andres R. H. Horie N. Slikker W. Keren-Gill H. Zhan K. Sun G. (2011). Human neural stem cells enhance structural plasticity and axonal transport in the ischaemic brain. *Brain* 134 1777–1789. 10.1093/brain/awr094 21616972 PMC3102243

[B6] Attwell D. Buchan A. M. Charpak S. Lauritzen M. Macvicar B. A. Newman E. A. (2010). Glial and neuronal control of brain blood flow. *Nature* 468 232–243. 10.1038/nature09613 21068832 PMC3206737

[B7] Bacigaluppi M. Pluchino S. Peruzzotti-Jametti L. Kilic E. Kilic U. Salani G. (2009). Delayed post-ischaemic neuroprotection following systemic neural stem cell transplantation involves multiple mechanisms. *Brain* 132 2239–2251. 10.1093/brain/awp174 19617198

[B8] Balseanu A. T. Buga A. M. Catalin B. Wagner D. C. Boltze J. Zagrean A. M. (2014). Multimodal approaches for regenerative stroke therapies: combination of granulocyte colony-stimulating factor with bone marrow mesenchymal stem cells is not superior to G-CSF alone. *Front. Aging Neurosci.* 6:130. 10.3389/fnagi.2014.00130 25002846 PMC4066299

[B9] Balusu S. Van Wonterghem E. De Rycke R. Raemdonck K. Stremersch S. Gevaert K. (2016). Identification of a novel mechanism of blood–brain communication during peripheral inflammation via choroid plexus derived extracellular vesicles. *EMBO Mol. Med.* 8 1162–1183. 10.15252/emmm.201606271 27596437 PMC5048366

[B10] Bang C. Thum T. (2012). Exosomes: new players in cell-cell communication. *Int. J. Biochem. Cell Biol.* 44 2060–2064. 10.1016/j.biocel.2012.08.007 22903023

[B11] Barile L. Vassalli G. (2017). Exosomes: therapy delivery tools and biomarkers of diseases. *Pharmacol. Ther.* 174 63–78. 10.1016/j.pharmthera.2017.02.020 28202367

[B12] Bellingham S. A. Guo B. B. Coleman B. M. Hill A. F. (2012). Exosomes: vehicles for the transfer of toxic proteins associated with neurodegenerative diseases? *Front. Physiol.* 3:124. 10.3389/fphys.2012.00124 22563321 PMC3342525

[B13] Burrello J. Monticone S. Gai C. Gomez Y. Kholia S. Camussi G. (2016). Stem cell-derived extracellular vesicles and immune-modulation. *Front. Cell Dev. Biol.* 4:83 10.3389/fcell.2016.00083 PMC4992732 27597941

[B14] Cai W. Liu H. Zhao J. Chen L. Y. Chen J. Lu Z. (2017). Pericytes in brain injury and repair after ischemic stroke. *Trans. Stroke Res.* 8 107–121. 10.1007/s12975-016-0504-4 27837475 PMC5350040

[B15] Caplan A. I. (2008). All MSCs are pericytes? *Cell Stem Cell* 3 229–230. 10.1016/j.stem.2008.08.008 18786406

[B16] Caplan A. I. (2016). MSCs: the sentinel and safe-guards of injury. *J. Cell. Physiol.* 231 1413–1416. 10.1002/jcp.25255 26565391

[B17] Caplan A. I. Correa D. (2011). The MSC: an injury drugstore. *Cell Stem Cell* 9 11–15. 10.1016/j.stem.2011.06.008 21726829 PMC3144500

[B18] Chen J. Chopp M. (2018). Exosome therapy for stroke. *Stroke* 49 1083–1090. 10.1161/STROKEAHA.117.018292 29669873 PMC6028936

[B19] Chen K.-H. Chen C.-H. Wallace C. G. Yuen C.-M. Kao G.-S. Chen Y.-L. (2016). Intravenous administration of xenogenic adipose-derived mesenchymal stem cells (ADMSC) and ADMSC-derived exosomes markedly reduced brain infarct volume and preserved neurological function in rat after acute ischemic stroke. *Oncotarget* 7 74537–74556. 10.18632/oncotarget.12902 27793019 PMC5342685

[B20] Cheng L. Zhang K. Wu S. Cui M. Xu T. (2017). Focus on mesenchymal stem cell-derived exosomes: opportunities and challenges in cell-free therapy. *Stem Cells Int.* 2017:6305295. 10.1155/2017/6305295 29410682 PMC5749272

[B21] Chivet M. Javalet C. Laulagnier K. Blot B. Hemming F. J. Sadoul R. (2014). Exosomes secreted by cortical neurons upon glutamatergic synapse activation specifically interact with neurons. *J. Extracell. Vesicles* 3:24722. 10.3402/jev.v3.24722 25398455 PMC4232649

[B22] Clarkson A. N. Huang B. S. Macisaac S. E. Mody I. Carmichael S. T. (2010). Reducing excessive GABA-mediated tonic inhibition promotes functional recovery after stroke. *Nature* 468 305–309. 10.1038/nature09511 21048709 PMC3058798

[B23] Cramer S. C. Wolf S. L. Adams H. P. Chen D. Dromerick A. W. Dunning K. (2017). Stroke recovery and rehabilitation research: issues, opportunities, and the National Institutes of Health StrokeNet. *Stroke* 48 813–819. 10.1161/STROKEAHA.116.015501 28174324 PMC5330812

[B24] Dammanahalli J. K. Wang X. Sun Z. (2011). Genetic interleukin-10 deficiency causes vascular remodeling via the upregulation of Nox1. *J. Hypertens.* 29 2116–2125. 10.1097/HJH.0b013e32834b22a0 21918473 PMC3373265

[B25] de Souza L. E. Malta T. M. Kashima Haddad S. Covas D. T. (2016). Mesenchymal stem cells and pericytes: to what extent are they related? *Stem Cells Dev.* 25 1843–1852. 10.1089/scd.2016.0109 27702398

[B26] Doeppner T. R. Bähr M. Giebel B. Hermann D. M. (2018). Immunological and non-immunological effects of stem cell-derived extracellular vesicles on the ischaemic brain. *Ther. Adv. Neurol. Disord.* 26:1756286418789326. 10.1177/1756286418789326 30083231 PMC6071165

[B27] Doeppner T. R. Ewert T. A. Tönges L. Herz J. Zechariah A. ElAli A. (2012). Transduction of neural precursor cells with TAT-Hsp70 chaperone: therapeutic potential against ischemic stroke after intrastriatal and systemic transplantation. *Stem Cells* 30 1297–1310. 10.1002/stem.1098 22593021

[B28] Doeppner T. R. Herz J. Görgens A. Schlechter J. Ludwig A.-K. Radtke S. (2015). Extracellular vesicles improve post-stroke neuroregeneration and prevent postischemic immunosuppression. *Stem Cells Transl. Med.* 4 1131–1143. 10.5966/sctm.2015-0078 26339036 PMC4572905

[B29] Doeppner T. R. Kaltwasser B. Bähr M. Hermann D. M. (2014a). Effects of neural progenitor cells on stroke impairment-detailed analysis of behavioral tests. *Front. Cell. Neurosci.* 8:338 10.3389/fncel.2014.00338 PMC4205824 25374509

[B30] Doeppner T. R. Kaltwasser B. Teli M. K. Bretschneider E. Bähr M. Hermann D. M. (2014b). Effects of acute versus post-acute systemic delivery of neural progenitor cells on neurological recovery and brain remodeling after focal cerebral ischemia in mice. *Cell Death Dis.* 5:e1386. 10.1038/cddis.2014.359 25144721 PMC4454329

[B31] Dozio V. Sanchez J. C. (2017). Characterisation of extracellular vesicle-subsets derived from brain endothelial cells and analysis of their protein cargo modulation after TNF exposure. *J. Extracell. Vesicles* 6:1302705. 10.1080/20013078.2017.1302705 28473883 PMC5405560

[B32] Fan G. C. (2014). Hypoxic exosomes promote angiogenesis. *Blood* 124 3669–3670. 10.1182/blood-2014-10-607846 25498451

[B33] Faure J. Lachenal G. Court M. Hirrlinger J. Chatellard-Causse C. Blot B. (2006). Exosomes are released by cultured cortical neurones. *Mol. Cell. Neurosci.* 31 642–648. 10.1016/j.mcn.2005.12.003 16446100

[B34] Frühbeis C. Fröhlich D. Kuo W. P. Krämer-Albers E. M. (2013). Extracellular vesicles as mediators of neuron-glia communication. *Front. Cell. Neurosci.* 7:182. 10.3389/fncel.2013.00182 24194697 PMC3812991

[B35] Gaceb A. Barbariga M. Özen I. Paul G. (2018). The pericyte secretome: potential impact on regeneration. *Biochimie* 10.1016/j.biochi.2018.04.015 [Epub ahead of print]. 29698670

[B36] Garcia J. M. Stillings S. A. Leclerc J. L. Phillips H. Edwards N. J. Robicsek S. A. (2017). Role of interleukin-10 in acute brain injuries. *Front. Neurol.* 8:244 10.3389/fneur.2017.00244 PMC5466968 28659854

[B37] Ginsberg M. D. (2008). Neuroprotection for ischemic stroke: past, present and future. *Neuropharmacology* 55 363–389. 10.1016/j.neuropharm.2007.12.007 18308347 PMC2631228

[B38] Goldie B. J. Dun M. D. Lin M. Smith N. D. Verrills N. M. Dayas C. V. (2014). Activity-associated miRNA are packaged in Map1b-enriched exosomes released from depolarized neurons. *Nucleic Acids Res.* 42 9195–9208. 10.1093/nar/gku594 25053844 PMC4132720

[B39] Goodenough D. A. Goliger J. A. Paul D. L. (1996). Connexins, connexons, and intercellular communication. *Annu. Rev. Biochem.* 65 475–502. 10.1146/annurev.bi.65.070196.002355 8811187

[B40] Hermann D. Buga A. M. Popa-Wagner A. (2015). Neurovascular remodeling in the aged ischemic brain. *J. Neural Transm.* 122 S25–S33. 10.1007/s00702-013-1148-0 24378703

[B41] Hermann D. M. Chopp M. (2012). Promoting brain remodelling and plasticity for stroke recovery: therapeutic promise and potential pitfalls of clinical translation. *Lancet Neurol.* 11 369–380. 10.1016/S1474-4422(12)70039-X 22441198 PMC3964179

[B42] Hermann D. M. ElAli A. (2012). The abluminal endothelial membrane in neurovascular remodeling in health and disease. *Sci. Signal.* 5:re4. 10.1126/scisignal.2002886 22871611

[B43] Hermann D. M. Zechariah A. (2009). Implications of vascular endothelial growth factor for postischemic neurovascular remodeling. *J. Cereb. Blood Flow Metab.* 29 1620–1643. 10.1038/jcbfm.2009.100 19654590

[B44] Holm M. M. Kaiser J. Schwab M. E. (2018). Extracellular vesicles: multimodal envoys in neural maintenance and repair. *Trends Neurosci.* 41 360–372. 10.1016/j.tins.2018.03.006 29605090

[B45] Koniusz S. Andrzejewska A. Muraca M. Srivastava A. K. Janowski M. Lukomska B. (2016). Extracellular vesicles in physiology, pathology, and therapy of the immune and central nervous system, with focus on extracellular vesicles derived from mesenchymal stem cells as therapeutic tools. *Front. Cell. Neurosci.* 10:109. 10.3389/fncel.2016.00109 27199663 PMC4852177

[B46] Krämer-Albers E. M. Hill A. F. (2016). Extracellular vesicles: interneural shuttles of complex messages. *Curr. Opin. Neurobiol.* 39 101–107. 10.1016/j.conb.2016.04.016 27183381

[B47] Kumar A. Stoica B. A. Loane D. J. Yang M. Abulwerdi G. Khan N. (2017). Microglial-derived microparticles mediate neuroinflammation after traumatic brain injury. *J. Neuroinflammation* 14:47. 10.1186/s12974-017-0819-4 28292310 PMC5351060

[B48] Lachenal G. Pernet-Gallay K. Chivet M. Hemming F. J. Belly A. Bodon G. (2011). Release of exosomes from differentiated neurons and its regulation by synaptic glutamatergic activity. *Mol. Cell. Neurosci.* 46 409–418. 10.1016/j.mcn.2010.11.004 21111824

[B49] Lai C. P.-K. Breakefield X. O. (2012). Role of exosomes/microvesicles in the nervous system and use in emerging therapies. *Front. Physiol.* 3:228. 10.3389/fphys.2012.00228 22754538 PMC3384085

[B50] Lener T. Gimona M. Aigner L. Börger V. Buzas E. Camussi G. (2015). Applying extracellular vesicles based therapeutics in clinical trials - an ISEV position paper. *J. Extracell. Vesicles* 4:30087. 10.3402/jev.v4.30087 26725829 PMC4698466

[B51] Li Y. Cheng Q. Hu G. Deng T. Wang Q. Zhou J. (2018). Extracellular vesicles in mesenchymal stromal cells: a novel therapeutic strategy for stroke. *Exp. Ther. Med.* 15 4067–4079. 10.3892/etm.2018.5993 29725359 PMC5920496

[B52] Ling H. Fabbri M. Calin G. A. (2013). MicroRNAs and other non-coding RNAsas targets for anticancer drug development. *Nat. Rev. Drug Discov.* 12 847–865. 10.1038/nrd4140 24172333 PMC4548803

[B53] Liu F. J. Lim K. Y. Kaur P. Sepramaniam S. Armugam A. Wong P. T. (2013). microRNAs involved in regulating spontaneous recovery in embolic stroke model. *PLoS One* 8:e66393. 10.1371/journal.pone.0066393 23823624 PMC3688919

[B54] Llorente A. van Deurs B. Sandvig K. (2007). Cholesterol regulates prostasome release from secretory lysosomes in PC-3 human prostate cancer cells. *Eur. J. Cell Biol.* 86 405–415. 10.1016/j.ejcb.2007.05.001 17601634

[B55] Lopatina T. Kalinina N. Karagyaur M. Stambolsky D. Rubina K. Revischin A. (2011). Adipose derived stem cells stimulate regeneration of peripheral nerves: BDNF secreted by these cells promotes nerve healing and axon growth *de novo*. *PLoS One* 6:e17899. 10.1371/journal.pone.0017899 21423756 PMC3056777

[B56] Mayo J. N. Bearden S. E. (2015). Driving the hypoxia inducible pathway in human pericytes promotes vascular density in an exosome dependent manner. *Microcirculation* 22 711–723. 10.1111/micc.12227 26243428 PMC4715585

[B57] Mosser D. M. Zhang X. (2008). Interleukin-10: new perspectives on an old cytokine. *Immunol. Rev.* 226 205–218. 10.1111/j.1600-065X.2008.00706.x 19161426 PMC2724982

[B58] Nakagomi T. Kubo S. Nakano-Doi A. Sakuma R. Lu S. Narita A. (2015). Brain vascular pericytes following ischemia have multipotential stem cell activity to differentiate into neural and vascular lineage cells. *Stem Cells* 33 1962–1974. 10.1002/stem.1977 25694098

[B59] Nakajima M. Nito C. Sowa K. Suda S. Nishiyama Y. Nakamura-Takahashi A. (2017). Mesenchymal stem cells overexpressing interleukin-10 promote neuroprotection in experimental acute ischemic stroke. *Mol. Ther. Methods Clin. Dev.* 6 102–111. 10.1016/j.omtm.2017.06.005 28725658 PMC5502709

[B60] Norden D. M. Fenn A. M. Dugan A. Godbout J. P. (2014). TGF beta produced by IL-10 redirected astrocytes attenuates microglial activation. *Glia* 62 881–895. 10.1002/glia.22647 24616125 PMC4061706

[B61] Otero-Ortega L. Gomez de Frutos M. C. Laso-Garcia F. Rodriguez-Frutos B. Medina-Gutierrez E. Lopez J. A. (2017). Exosomes promote restoration after an experimental animal model of intracerebral haemorrhage. *J. Cereb. Blood Flow Metab.* 38 767–779. 10.1177/0271678X17708917 28524762 PMC5987932

[B62] Popa-Wagner A. Buga A. M. Kokaia Z. (2011). Perturbed cellular response to brain injury during aging. *Aging Res. Rev.* 10 71–79. 10.1016/j.arr.2009.10.008 19900590

[B63] Popa-Wagner A. Carmichael S. T. Kokaia Z. Walker L. C. (2007). The response of the aged brain to stroke: too much, too soon? *Curr. Neurovasc. Res.* 4 216–277. 10.2174/156720207781387213 17691975

[B64] Popa-Wagner A. Dinca I. Yalikun S. Walker L. Kroemer H. Kessler C. (2006). Accelerated delimitation of the infarct zone by capillary-derived nestin-positive cells in aged rats. *Curr. Neurovasc. Res.* 3 3–13. 10.2174/156720206775541732 16472121

[B65] Popa-Wagner A. Glavan D. G. Olaru A. Olaru D. G. Margaritescu O. Tica O. (2018). Present status and future challenges of new therapeutic targets in preclinical models of stroke in aged animals with/without comorbidities. *Int. J. Mol. Sci.* 19:E356. 10.3390/ijms19020356 29370078 PMC5855578

[B66] Potolicchio I. Carven G. J. Xu X. Stipp C. Riese R. J. Stern L. J. (2005). Proteomic analysis of microglia-derived exosomes: metabolic role of the aminopeptidase CD13 in neuropeptide catabolism. *J. Immunol.* 175 2237–2243. 10.4049/jimmunol.175.4.2237 16081791

[B67] Record M. Poirot M. Silvente-Poirot S. (2014). Emerging concepts on the role of exosomes in lipid metabolic diseases. *Biochimie* 96 67–74. 10.1016/j.biochi.2013.06.016 23827857

[B68] Reitmeir R. Kilic E. Kilic U. Bacigaluppi M. ElAli A. Salani G. (2011). Post-acute delivery of erythropoietin induces stroke recovery by promoting perilesional tissue remodelling and contralesional pyramidal tract plasticity. *Brain* 134 84–99. 10.1093/brain/awq344 21186263

[B69] Rosell A. Lo E. H. (2008). Multiphasic roles for matrix metalloproteinases after stroke. *Curr. Opin. Pharmacol.* 8 82–89. 10.1016/j.coph.2007.12.001 18226583

[B70] Salido-Guadarrama I. Romero-Cordoba S. Peralta-Zaragoza O. Hidalgo-Miranda A. Rodríguez-Dorantes M. (2014). MicroRNAs transported by exosomes in body fluids as mediators of intercellular communication in cancer. *Onco Targets Ther.* 7 1327–1338. 10.2147/OTT.S61562 25092989 PMC4114916

[B71] Savitz S. I. Fisher M. (2007). Future of neuroprotection for acute stroke: in the aftermath of the SAINT trials. *Ann. Neurol.* 61 396–402. 10.1002/ana.21127 17420989

[B72] Skotland T. Sandvig K. Llorente A. (2017). Lipids in exosomes: current knowledge and the way forward. *Prog. Lipid Res.* 66 30–41. 10.1016/j.plipres.2017.03.001 28342835

[B73] Strauss K. Goebel C. Runz H. Möbius W. Weiss S. Feussner I. (2010). Exosome secretion ameliorates lysosomal storage of cholesterol in niemann-pick type c disease. *J. Biol. Chem.* 285 26279–26288. 10.1074/jbc.M110.134775 20554533 PMC2924046

[B74] Taylor A. R. Robinson M. B. Gifondorwa D. J. Tytell M. Milligan C. E. (2007). Regulation of heat shock protein 70 release in astrocytes: role of signaling kinases. *Dev. Neurobiol.* 67 1815–1829. 10.1002/dneu.20559 17701989

[B75] Théry C. Boussac M. Véron P. Ricciardi-Castagnoli P. Raposo G. Garin J. (2001). Proteomic analysis of dendritic cell-derived exosomes: a secreted subcellular compartment distinct from apoptotic vesicles. *J. Immunol.* 166 7309–7318. 10.4049/jimmunol.166.12.7309 11390481

[B76] Todorova D. Simoncini S. Lacroix R. Sabatier F. Dignat-George F. (2017). Extracellular vesicles in angiogenesis. *Circ. Res.* 120 1658–1673. 10.1161/CIRCRESAHA.117.309681 28495996 PMC5426696

[B77] Tsenkina Y. Ricard J. Runko E. Quiala-Acosta M. M. Mier J. Liebl D. J. (2015). EphB3 receptors function as dependence receptors to mediate oligodendrocyte cell death following contusive spinal cord injury. *Cell Death Dis.* 6:e1922. 10.1038/cddis.2015.262 26469970 PMC4632292

[B78] Valadi H. Ekström K. Bossios A. Sjöstrand M. Lee J. J. Lötvall J. O. (2007). Exosome-mediated transfer of mRNAs and microRNAs is a novel mechanism of genetic exchange between cells. *Nat. Cell Biol.* 9 654–659. 10.1038/ncb1596 17486113

[B79] Van Meer G. Voelker D. R. Feigenson G. W. (2008). Membrane lipids: where they are and how they behave. *Nat. Rev. Mol. Cell Biol.* 9 112–124. 10.1038/nrm2330 18216768 PMC2642958

[B80] Verderio C. Muzio L. Turola E. Bergami A. Novellino L. Ruffini F. (2012). Myeloid microvesicles are a marker and therapeutic target for neuroinflammation. *Ann. Neurol.* 72 610–624. 10.1002/ana.23627 23109155

[B81] Von Bartheld C. S. Altick A. L. (2011). Multivesicular bodies in neurons: distribution, protein content, and trafficking functions. *Prog. Neurobiol.* 93 313–340. 10.1016/j.pneurobio.2011.01.003 21216273 PMC3055956

[B82] Xin H. Katakowski M. Wang F. Qian J.-Y. Shuang Liu X. Ali M. M. (2017a). MiR-17-92 cluster in exosomes enhance neuroplasticity and functional recovery after stroke in rats. *Stroke* 48 747–753. 10.1161/STROKEAHA.116.015204 28232590 PMC5330787

[B83] Xin H. Wang F. Li Y. Lu Q.-E. Cheung W. L. Zhang Y. (2017b). Secondary release of exosomes from astrocytes contributes to the increase in neural plasticity and improvement of functional recovery after stroke in rats treated with exosomes harvested from MicroRNA 133b-overexpressing multipotent mesenchymal stromal cells. *Cell Trans.* 26 243–257. 10.3727/096368916X693031 27677799 PMC5303172

[B84] Xin H. Li Y. Cui Y. Yang J. J. Zhang Z. G. Chopp M. (2013). Systemic administration of exosomes released from mesenchymal stromal cells promote functional recovery and neurovascular plasticity after stroke in rats. *J. Cereb. Blood Flow Metab.* 33 1711–1715. 10.1038/jcbfm.2013.152 23963371 PMC3824189

[B85] Yang S. Jin H. Zhu Y. Wan Y. Opoku E. N. Zhu L. (2017). Diverse functions and mechanisms of pericytes in ischemic stroke. *Curr. Neuropharmacol.* 15 892–905. 10.2174/1570159X15666170112170226 28088914 PMC5652032

[B86] Zhang Z. G. Chopp M. (2016). Exosomes in stroke pathogenesis and therapy. *J. Clin. Invest.* 126 1190–1197. 10.1172/JCI81133 27035810 PMC4811130

[B87] Zlokovic B. V. (2008). The blood–brain barrier in health and chronic neurodegenerative disorders. *Neuron* 57 178–201. 10.1016/j.neuron.2008.01.003 18215617

